# Keel bone fractures induce a depressive-like state in laying hens

**DOI:** 10.1038/s41598-020-59940-1

**Published:** 2020-02-20

**Authors:** E. A. Armstrong, C. Rufener, M. J. Toscano, J. E. Eastham, J. H. Guy, V. Sandilands, T. Boswell, T. V. Smulders

**Affiliations:** 1 Centre for Behaviour & Evolution, Newcastle University, Newcastle upon Tyne, UK; 2 Biosciences Institute, Newcastle University, Newcastle upon Tyne, UK; 3 Department of Animal Science, University of California, Davis, USA; 4 Centre for Proper Housing: Poultry and Rabbits (ZTHZ), University of Bern, Zollikofen, Switzerland; 5 School of Natural and Environmental Sciences, Newcastle University, Newcastle upon Tyne, UK; 6 Department of Agriculture, Horticulture and Engineering Sciences, SRUC, Edinburgh, UK

**Keywords:** Limbic system, Adult neurogenesis, Stress and resilience

## Abstract

In commercial flocks of laying hens, keel bone fractures (KBFs) are prevalent and associated with behavioural indicators of pain. However, whether their impact is severe enough to induce a depressive-like state of chronic stress is unknown. As chronic stress downregulates adult hippocampal neurogenesis (AHN) in mammals and birds, we employ this measure as a neural biomarker of subjective welfare state. Radiographs obtained longitudinally from Lohmann Brown laying hens housed in a commercial multi-tier aviary were used to score the severity of naturally-occurring KBFs between the ages of 21–62 weeks. Individual birds’ transitions between aviary zones were also recorded. Focal hens with severe KBFs at 3–4 weeks prior to sampling (n = 15) had lower densities of immature doublecortin-positive (DCX^+^) multipolar and bipolar neurons in the hippocampal formation than focal hens with minimal fractures (n = 9). KBF severity scores at this time also negatively predicted DCX^+^ cell numbers on an individual level, while hens that acquired fractures earlier in their lives had fewer DCX^+^ neurons in the caudal hippocampal formation. Activity levels 3–4 weeks prior to sampling were not associated with AHN. KBFs thus lead to a negative affective state lasting at least 3–4 weeks, and management steps to reduce their occurrence are likely to have significant welfare benefits.

## Introduction

Keel bone fractures (KBFs) present a serious welfare problem for the egg production industry, given that between 20 to 96% of birds within commercial flocks in various countries are reported to have some level of damage (Belgium^1^; Canada^2^; Denmark^3^; The Netherlands^4^; Switzerland^5,6^; and the UK^7–9^). Estimates of KBF prevalence increase with age, rising from 5.5% of birds affected within a flock at onset of lay^10^ to as many as 97% by the end of a production cycle^4^. Many KBFs appear to arise from collisions, both with perches^11^, which hens are highly motivated to use^12^, and other equipment including drinkers and support beams. Although the presence of a KBF does not suppress perching behaviour^13^, it does reduce frequency of range access via popholes^14^. Further behavioural evidence also supports the assumption that KBFs are a source of pain or discomfort for laying hens (reviewed by Riber *et al*.^15^), with recent studies demonstrating their association with altered movement throughout the aviary^16,17^. Focusing on more direct assessments of movement and pain, hens with KBFs display greater latencies to fly down from perches 100 and 150 cm above the floor to obtain a food reward than hens without fractures, whilst delays in fractured birds alone are reduced following administration of various analgesics^18–20^. Furthermore, a conditioned place preference for the location in which the analgesic was administered is observed to develop only in hens with KBFs^21^. While this indicates that short-term relief from pain arising from keel fractures is a reinforcing occurrence, whether their un-medicated experience is negative and/or salient enough to produce a chronic state of stress is unknown. Stress constitutes an integrated response to real or perceived threats to homeostasis or well-being^22^, but, when prolonged, can have deleterious consequences for health and welfare^23^, including symptoms of a depressive state. Physiological consequences include immunosuppression^24^ and delayed or reduced egg production^25^. Chronic stress thus has significant implications for commercial profitability, in addition to welfare concerns. Furthermore, whether pain arising from KBFs has an affective component for chickens, in addition to sensory/nociceptive components, has yet to be explored.

Hippocampal measures of long-term stress have a high level of validity (reviewed by Poirier *et al*.^26^). In mammals, the process of adult hippocampal neurogenesis (AHN) involves the production of new neurons (granule cells) which are integrated into the dentate gyrus subfield of the hippocampal formation (HF)^27,28^, and is sensitive to environmental conditions and prolonged experiences^29,30^. Specifically, AHN in rats and mice is downregulated following chronic exposure to stress^31^ or pain^32^, but stimulated by long term positive experiences, such as environmental enrichment^33^, voluntary exercise^34,35^ and antidepressant treatment^36,37^. Given that the AHN process co-varies with experience in a valence-dependent direction, it may provide a biomarker which reflects the subjective affective state of an animal^26^.

Evidence also supports the use of AHN as a proxy for the emotional component of stress. For example, AHN levels are restored by an antidepressant agent only in individual rats that also show behavioural anhedonia recovery, as quantified by increased sucrose intake^38^. A causal role for AHN in the behavioural recovery from induced stress afforded by antidepressant treatment^39,40^ or transfer to an enriched environment^41^ has also been demonstrated, indicating its reliable concurrence with changing mood state.

Accumulating evidence suggests that AHN levels in birds respond to similar environmental factors as in mammals. Housing in an enriched environment^42^ and opportunities to store and retrieve food caches^43,44^ upregulate avian AHN, though it is not possible to distinguish the potential contribution of positive affective responses to these conditions from their stimulation of hippocampal spatial processing mechanisms. However, avian AHN is also suppressed by various forms of chronic stress. Firstly, a group of studies in chickadees (*Poecile atricapillus & gambeli*) demonstrate that AHN is reduced in wild-caught birds placed in captivity^43,45^, but levels in hand-reared birds are comparable to free-living conspecifics^46^. This suggests that down-regulated AHN in the former group is attributable to the stress of transition to captive conditions, rather than reduced cognitive stimulation in the relatively impoverished captive environment^47^. Secondly, commercial broiler breeders subject to food restriction display lower AHN levels than counterparts fed *ad libitum*^48^, whilst AHN is suppressed following two weeks of constant light exposure in diurnal Indian house crows *Corvus splendens*^49^. Finally, laying hens exposed to 8 weeks of unpredictable chronic mild stress show selective loss of new neurons at the caudal HF pole^50^. AHN in several avian species thus appears responsive to various forms of chronic stress.

According to a functional gradient across the longitudinal axis of the mammalian HF^51^, the dorsal region (in rodents, posterior in primates) is more heavily implicated in spatial cognition^52–54^, whereas the ventral region (anterior in primates) predominantly coordinates emotional modulation^55^. Correspondingly, reductions in AHN caused by chronic stress occur selectively at the ventral hippocampal pole in certain rodent models^29,38,56–58^. Though the avian HF differs from its mammalian homologue in cytoarchitecture^59^, neuroanatomy leads us to hypothesise that the caudal avian HF may be homologous to the ventral rodent subregion^60^. Indeed, electrical stimulation of the caudal pigeon (*Columba livia domestica*) HF has a greater suppressive effect upon activity of the hypothalamic-pituitary-adrenal stress axis than stimulation at more rostral sites^61^, pointing to a similarly greater role in affective modulation. AHN in the caudal subregion is also more sensitive to unpredictable chronic mild stress in laying hens^50^. Thus, factors driving long-term stress in poultry may have a stronger influence on AHN at the caudal HF pole.

Evidence suggests that prolonged exposure to pain is sufficient to induce measurable chronic stress in the rodent brain. Indeed, a model of neuropathic pain in mice resulted in marked reductions in AHN, accompanied by behavioural indicators of anxiety- and depressive-like states^32^. Moreover, these neurogenic and behavioural changes persisted beyond reversal of the injury, indicating their independence from nociceptive stimulation. The pain stimulus thus resulted in measurable chronic stress that may have been associated with a prolonged negative subjective state. If KBFs in laying hens present a comparable pain stimulus, then individual differences in acquired severity within commercial housing units may translate to varying degrees of chronic stress for hens. This has clear implications for welfare management. To test this hypothesis, we sampled hens at two extremes of the KBF spectrum within a commercially-relevant aviary housing system, namely those with minimal and severe fractures, after collecting longitudinal data on individual fracture severity scores. To quantify the number of surviving new-born cells generated through AHN, we stained sections for doublecortin (DCX), an endogenous protein marker of migratory immature neurons in the mammalian^62^ and avian^63^ brain. We predicted that hens with severe KBFs would have a lower density of DCX-positive cells in the HF than counterparts with minimal KBFs, with the effect potentially more pronounced at the caudal HF pole. In addition, a linear relationship across hens was predicted, whereby individuals with greater KBF severity scores were expected to have a relatively lower density of new-born hippocampal cells. Finally, since pain arising from KBFs might be expected to reduce hen’s mobility, and voluntary exercise is known to linearly predict AHN in rodents^64^, it was necessary to account for a potential confounding effect of activity on AHN levels. An infra-red tracking system was thus used to record the number of transitions that individual hens made between aviary zones. Whether this measure was associated with DCX^+^ cell counts - either independent of, or in relation to, KBF scores - was also explored.

## Methods

### Ethical statement

Ethical approval for the study was obtained from the Veterinary Office of the Canton of Bern in Switzerland (approval number BE31/15). The experiment complied with Swiss regulations regarding the treatment of experimental animals.

### Animals & housing

One day old Lohmann Brown (LB) and Lohmann Selected Leghorn (LSL) chicks arrived at the Aviforum research facility in Zollikofen, Switzerland, where they were housed within eight mixed hybrid rearing pens. The study reported here was part of a larger effort, the facilities and management protocols of which are described in full by Rufener *et al*.^16^. At 18 weeks of age, hens were transferred to a commercial aviary system for laying hens within a layer barn on site. The layer barn was divided into 20 identical pens (450 × 700 × 230 cm), each holding 225 hens. In each of three pens, 20 focal LB hens were co-housed with 205 LSL hens of the same age. The minority LB hens within each pen all came from the same rearing pen. The differing phenotype of the white LSL hens facilitated the identification and catching of focal LB hens throughout the study. Three other pens in the barn contained focal LSL hens with a majority of LB hens, but as this strain was not sampled for quantification of AHN, their results are not reported here (see Rufener *et al*.^16^ for mobility data). In addition to spiral leg rings colour-coded for pen (Fieger AG, Untertuttwil, Switzerland), focal hens were marked with flexible legbands (Roxan Developments Ltd, Selkirk, United Kingdom) displaying individual identification numbers. Hens were left for three weeks to habituate to the new housing environment before mobility tracking commenced.

Each pen of the layer barn was equipped with a commercial aviary system (Bolegg Terrace, Vencomatic; Krieger AG, Ruswil, Switzerland). The system consisted of three tiers: (1) a lower tier (73 cm above ground), (2) a nest box tier with integrated group nests (153 cm above ground) and (3) a top tier (220 cm above ground), and was subject to minor modifications from the standard installed model. Specifically, the perch on the top tier was removed and one drinker line was moved from in front of the nest box to the top tier. Food and water were provided *ad libitum* through automatic feeding chains and nipple drinkers on the top and lower tier (with feed refreshed every two hours during light hours). Artificial light was provided from 02:00 h until 17:00 h, with a 5 minute dawn (02:00 h–02:05 h) and a 30 minute dusk (16:30 h–17:00 h) phase. Natural daylight was managed via curtains in front of the windows, which were open from 08:00 h until 16:00 h. Perches consisted of round metal rails (diameter: 3.2 cm, length: 230 cm) located on the top tier for roosting (six perches at 270 cm height, two perches at 300 cm height) and across the system to facilitate movement between tiers (three perches at 190 cm and 125 cm above ground on each side of the aviary, on top of the feeder on the lower tier). In total, 14 cm of perch space per hen was provided. The floor besides and underneath the aviary was covered with wood shavings (approx. 10 cm deep). Stocking density was 7.4 hens/m^2^ of accessible floor space, which included the littered floor area and all mesh grid areas in the lower and top tiers. Pecking opportunities were provided in the form of autoclaved aerated concrete stones (Xella Porenbeton Schweiz AG, Zurich, Switzerland) and *ad libitum* mineralized pecking stones (FORS 228 Pickschale Geflügel; Kunz Kunath AG, Burgdorf, Switzerland). To increase opportunities for explorative behaviour (scratching, pecking), straw was supplied in racks placed in the litter area. The wintergarden provided a covered area (9.32 m^2^) external to each pen, containing wood shavings and a dust bathing area filled with sand, and was accessible via popholes (15 cm above ground level) which opened automatically at 10:00 h and were closed manually between 16:00 and 16:30 h.

### Data collection

Data were collected at 11 time points during the production cycle. For one pen (20 focal birds), this occurred when the birds were 21, 24, 27, 31, 35, 39, 44, 48, 52, 57, and 61 weeks of age. For practical reasons, data from the remaining two pens (40 focal birds) were collected the subsequent week, i.e., 22, 25, 28, 32, 36, 40, 45, 49, 53, 58, and 62 weeks of age. Data on individual mobility were collected for six days per time point, and on the final day, hens were radiographed to detect fractures.

### Individual mobility

Individual mobility was recorded using a custom-made infrared (IR) tracking system which has been previously described and validated^65^. Infrared emitters were installed on the vertical grid panels dividing pens and generated infrared beams encoded with specific signals for the five pre-defined zones: litter, lower tier, nest box, upper tier, and wintergarden. Infrared receivers were mounted on the legbands of focal hens and recorded the zone-specific signals produced by the IR emitters with a frequency of 1 Hz, along with the date and time of each zone change. The system therefore recorded vertical transitions made across the aviary, but did not track horizontal movement within zones. Receivers were covered by a small plastic container to protect from dust, moisture and faeces (outer diameter: 3.1 cm, height: 2 cm; Semadeni AG, Ostermundigen, Switzerland) throughout the experiment. Containers were replaced if they became opaque due to dirt or scratches. Equipment mounted on the hens weighed 9.4 g, well below the suggested limit of 5% of body mass^66^.

At each of the 11 study time points, hens were caught and equipped with IR receivers on the day preceding mobility data collection (day 1). This allowed time for both habituation to the additional weight of the receiver and re-establishment of normal mobility behaviour after handling, given this experience has been shown to impact tonic immobility behaviour occurring directly afterwards^67^. Monitoring devices have been found to reduce exploration by adult hens on the day of fitting, but to have negligible effects on behaviour from two days onwards^68^. Mobility data were therefore collected from day 2 to day 7, before receivers were removed and data were downloaded as CSV files on day 8.

### Keel bone assessment/fracture severity scoring

At each time point, after removing the IR receiver on day 8, hens were radiographed to detect keel bone fractures using a mobile X-ray unit (GIERTH HF 200 ML; x-ray tube Toshiba D-124 with maximal acceleration voltage of 100 kV; x-ray plate Canon CXDI-50G; software Canon CXDI Control Software NE; distance: 80 cm, voltage: 46 kV/2.4 mAs). Hens were firmly held by both legs, carefully turned upside down and fixated in padded metal shackles to induce immobility^69^. The procedure took approximately 10–20 seconds per radiograph and occurred 11 times (T1–T11) throughout each hen’s lifetime. In a previous study, KBF severity at 61 weeks did not differ between repeatedly-radiographed focal hens and non-focal hens radiographed only at this time, indicating that multiple radiographs had no negative impact on keel integrity^70^. Though focal LSL hens produced fewer eggs, it is not clear if this related to stress of the radiograph procedure or of being the minority hybrid, whilst egg production in focal LB hens (used in the present study) was not affected^70^.

Radiographs were imported to the PACS (Picture Archiving and Communication System; IMPAX EE, Agfa Healthcare, Bonn, Germany) of the Department of Clinical Radiology (Vetsuisse Faculty, University of Bern) as DICOM files. For subsequent analysis, radiographs were downloaded from the PACS as JPEG files.

Radiographs were analyzed according to aggregate fracture severity and the presence of a visible fracture gap. The observer was blind to both the age and identity of the hen. Aggregate fracture severity was assessed using a tagged visual analogue scale ranging from “no fracture” to “extremely severe”, resulting in a continuous variable ranging from 0.0 to 10.0. The system and its validation is described in detail by Rufener *et al*.^71^. Eleven days after final radiographs were conducted, preliminary analysis of processed radiographs up to the penultimate time point (T10) was used to select 12 birds with minimal KBFs and 12 birds with severe KBFs for brain sampling. This sample size previously afforded sufficient power to detect an effect of chronic stress on the same AHN outcome measure in laying hens^50^. It was not possible to select a control group of hens with no fractures, as although these were very minor in some cases, birds entirely free of KBFs did not exist by the end of the study. The higher proportion of KBFs detected here compared to previous studies may relate to the sensitivity of the relatively novel radiography technique, compared to less reliable methods such as palpation^71^.

### Tissue collection & processing

Following a delay of 3.5 (pen 1) or 4.5 (pens 2 & 3) weeks since the final x-ray, focal hens (n = 24) were killed via an intravenous injection of pentobarbital (Esconarkon, 0.3 ml/hen). Immediately thereafter, brains were removed from the skull, placed into 0.1 M PBS in a Petri dish and divided along the longitudinal fissure with a scalpel. The hippocampus from one hemisphere was allocated for molecular biology, with results to be reported elsewhere. The remaining hemisphere from each brain was immersion fixed for 44–48 h in 4% paraformaldehyde in 0.5 M Phosphate Buffered Saline (PFA - PBS) at 4 °C in preparation for immunohistochemistry. To balance possible lateralisation of AHN and its responsivity, tissue collected for each purpose was alternated between the left and right hemisphere within the minimal and severe KBF groups. Samples were then cryoprotected in a solution of 30% sucrose in 0.5 M PBS, before being embedded in OCT (4583, Electron Microscopy Sciences - USA). Coronal sections (50 μm) were cut on a cryostat (HM 550, Microm – Germany) and stored in cryoprotectant solution (30% glycerol, 30% ethylene glycol, 0.1M PBS) at −20 °C. Serial sections taken at 400μm intervals were processed for immunohistochemistry.

### Immunohistochemistry

To quantify the number of surviving newborn cells generated through AHN, sections were stained for doublecortin (DCX), an endogenous protein marker of migratory immature neurons in the avian brain^63^. Exercise upregulates proliferation in the mammalian hippocampus, and a sustained effect of running on subsequent newborn cell survival has been reported to persist for five weeks after discontinuation of this exercise in mice^72^. Since DCX is expressed for approximately 4 weeks from the start of neuronal differentiation^63^, activity levels at the most recent time-point (~3–4 weeks prior) were those most likely to relate to survival of the stained cell population present at the time of death, and were thus analysed in relation to AHN. Staining was conducted over 6 batches, each containing tissue from 4 birds, balanced for KBF status. Free-floating tissue slices were washed in 0.1 M PBS to remove cryoprotectant (3 × 5 mins), then underwent 30 minutes endogenous peroxidase inhibition in 1% H_2_O_2_ (Sigma-Aldrich, UK). Samples were again washed in PBS before 60 minutes incubation in blocking solution, containing 2% normal goat serum and 1% Bovine Serum Albumin [BSA] dissolved in 0.1 M PBS that contained 0.3% Triton X-100. After a quick rinse in distilled H_2_O, samples were incubated overnight in rabbit polyclonal to doublecortin primary antibody (Abcam Cat# ab18723, RRID:AB_732011) at concentration 1:1000 (4 °C). Following washes in PBS, samples were incubated for 120 minutes at room temperature in 1:500 biotinylated anti-rabbit IgG secondary antibody, made in goat (Vector Labs, BA-1000). Samples were washed in PBS before conjugate enzyme incubation in 1:250 Horse Radish Streptavidin (Vector Labs, SA-5004) for 60 minutes. Following washes in PBS and dH_2_O, 30 seconds chromogen incubation in 3,3′-Diaminobenzidine (DAB) was conducted by diluting SIGMAFAST tablets in pure water to final concentration of 0.35 mg/ml. Tissue was rinsed immediately in dH_2_O to stop the reaction. Following final washes in 0.1 M PBS, slices were mounted onto gelatine-subbed slides using a paintbrush in dH_2_O. Once dry, slides were soaked for 2 × 5 minutes in Histoclear before coverslipping using Eukitt® (03989 FLUKA). Excess mounting medium was cleaned from the slides using a razor blade after drying.

### Quantification of AHN

For every animal, 4 to 6 hippocampal sections 800 μm apart were systematically analysed, starting with the rostral-most section bearing hippocampal tissue. This sampling spanned roughly 1/16^th^ of the rostral and 1/8^th^ to 1/4^th^ of the caudal HF, given the latter region curls around the back of the forebrain and is thus contained within fewer coronal slices. Hippocampal slices were analysed with an optical microscope (Leica DM6B-Z, Germany) equipped with a digital video camera (Leica DFC450 C, Germany) and motorized stage system (Leica AHM, Germany) to step through sections for systematic sampling. Quantification was performed by a single observer (EA), blind to the KBF status of the animals.

Image analysis was performed with Stereo Investigator software (version 2018.1.1, MBF Bioscience, USA). Hippocampal borders were outlined at 2.5X magnification (0.07 numerical aperture) on every analysed slice according to the chick stereotaxic atlas^73^. Because of the complex structure of the avian HF, we divided the whole structure in two major components: i) the rostral hippocampus (RH - interaural 5.68/0.50) and ii) the caudal hippocampus (CH - interaural 0.50/−0.50). Cell counting was performed at 100X magnification (0.65 numerical aperture). Stereological parameters were set to an optical fractionator grid of 120 × 120 µm for RH and 240 × 240 µm for CH, a counting frame of 50 × 50 µm for both regions and a mounted thickness of 20 µm.

Although no granule cells are present in the avian HF, different types of DCX^+^ cells have been previously described in avian brain literature^74^ and can be divided into two groups according to neuronal morphology: (I) multipolar neurons and (II) bipolar (fusiform) cells. In line with Boseret *et al*.^74^, we assume that the fusiform neurons are younger and still migrating, while the multipolar neurons are more mature and settling. Multipolar cells were defined as medium-large sized cells, with a round or polygonal/angular cell body shape and process branching from it in three or more directions. Golgi analysis of the chick HF^75^ suggests that this group includes a high proportion of multipolar projection neurons, but may also incorporate pyramidal and multipolar local circuit neurons. Bipolar/fusiform cells were defined as medium-small sized cells with elliptical or oval cell body shape and process branching from it in two or fewer directions. Cells of these two types lying inside the optical fractionator frame or bisected by its green lines were counted, according to the Optical Fractionator method.

Counts were exported to MS Excel from Stereo Investigator and used to manually calculate densities of each cell type. Specifically, the number of counted cells of each type was divided by the area of the counting frame (2500 µm), multiplied by both the number of counting sites sampled in that brain and the section thickness (50 µm), to produce a density per volume measure for the sampled tissue. Values were transformed to densities per cubic millimetre by multiplying by 10^9^. The rostral and caudal hippocampus were treated separately.

### Statistical analysis

When analysing radiographs from the final time point (T11) after tissue collection, it was necessary to move 3 sampled birds from the minimal to the severe KBF group for the purpose of statistical analysis. Final sample sizes for between-group analyses were thus 9 minimal KBF and 15 severe KBF hens. Descriptive statistics are displayed as mean (M) ± standard deviation (SD), and all analyses were run in IBM SPSS Statistics (v24). To compare activity of the minimal and severe KBF groups at time point 1 (before most fractures occurred) and time point 11, a linear mixed model (LMM) was conducted for the mean aviary transitions made during the 6 days preceding each. Experimental pen was included as a random factor, with time point as a repeated fixed factor and final KBF severity group as a between-subject fixed factor. To assess the relationship between KBF severity and AHN, separate LMMs were conducted for raw densities of DCX-expressing multipolar and bipolar cells. In all models, staining batch and experimental pen were included as random factors, whilst HF subregion (rostral/caudal) was included as a repeated fixed factor. When exploring group differences, fracture status at the final time point (minimal/severe KBF) was included as a between-subject fixed factor, and in an interaction term with HF subregion. To assess within-individual relationships, KBF severity score at the final time point (T11) and the mean number of transitions made between aviary zones over the preceding 6 days were entered as covariates in separate models, each of which included their interaction with HF subregion. To determine which variable best predicted cell densities, a model including both covariates (and their interactions with subregion) was also run. Several analyses were conducted in order to explore the influence of the timescale of KBF acquisition on AHN at the end of life. Firstly, the time point at which a hen suffered its first KBF was included as a covariate in LMMs for the two cell types, again alongside staining batch and pen as random factors, HF subregion as a fixed factor and the interaction between time point and subregion. Secondly, to determine at which time point fracture severity best predicted DCX^+^ cell densities, and if severity at any other times added explanatory power, LMMs including severity score at each time as a covariate were conducted in a stepwise forward manner. Lastly, the changes in KBF severity compared to the previous recorded score at each time point were included as covariates in LMMs, according to the same stepwise procedure. These analyses were conducted separately for DCX^+^ multipolar and bipolar densities. All figures display cell densities normalised (z-scored) within their staining batch.

## Results

Subsequent to readjusting sample groups based on KBF severity in radiographs taken at the final time point, hens in the minimal KBF group (n = 9) had a mean fracture severity score of 3.5 (±0.87 SD), with individual scores ranging from 1.9–4.8. In contrast, hens in the severe KBF group (n = 15) had a mean final fracture severity of 8.5 (±0.85 SD) and a range of 7.2–10.0.

### Changes in activity levels

**Figure 1:**
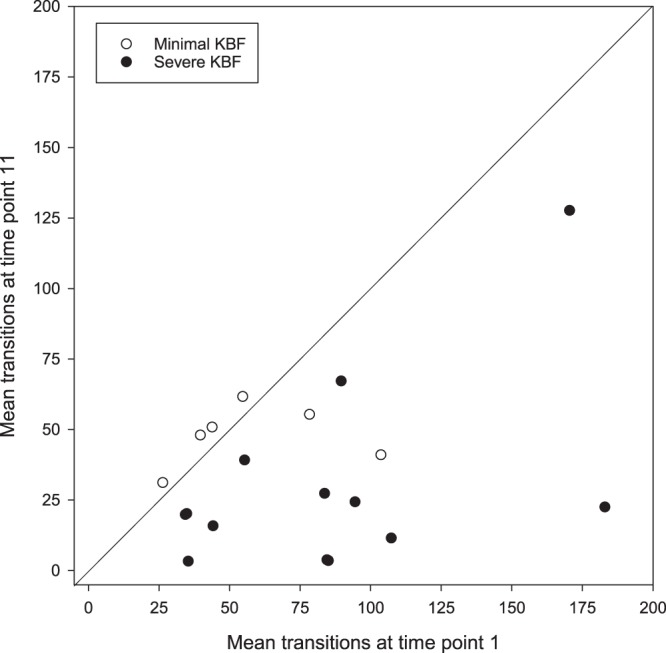
The mean number of vertical aviary transitions made by hens which had developed minimal versus severe keel bone fractures (KBFs) by time point 11, made during the 6 days preceding the first (T1) and final (T11) radiograph time points. Line of equality represents equal activity at both time points. Some data points are overlapping.

The mean number of aviary transitions made during the 6 days preceding the first and last study time points (T1 and T11) was compared for hens who had developed minimal versus severe KBFs by the latter time point (see Fig. 1). Due to some loss of signal, transitions at both of these time points were successfully recorded for 6 minimal and 13 severe KBF hens. At time point 1, all hens in the minimal KBF group had severity scores of 0, which was also the case for all but two individuals in the severe KBF group (scoring 3.0 and 4.0). The KBF groups did not differ in their overall vertical transitions (*F*_1,22.2_ = 0.010, *p* = 0.921), whilst the number of transitions made by all hens decreased between time point 1 (M = 75.0 ± 40.1 SD) and time point 11 (M = 35.5 ± 29.7 SD, *F*_1,21.5_ = 14.35, *p* = 0.001). An interaction existed between study time point and KBF severity group (*F*_1,21.5_ = 5.996, *p* = 0.023). Whereas there was no difference in the number of transitions made by minimal KBF birds at T1 (M = 60.40 ± 24.5 SD) and T11 (M = 48.0 ± 10.8 SD; *p* = 0.422), severe KBF birds made fewer transitions at T11 (M = 29.7 ± 34.1 SD) than they had at T1 (M = 82.8 ± 45.1 SD; *p* < 0.001). Minimal and severe KBF birds did not differ from each other in the absolute number of transitions made at either T1 (*p* = 0.209) or T11 (*p* = 0.169).

### Adult hippocampal neurogenesis & KBF severity group

**Figure 2:**
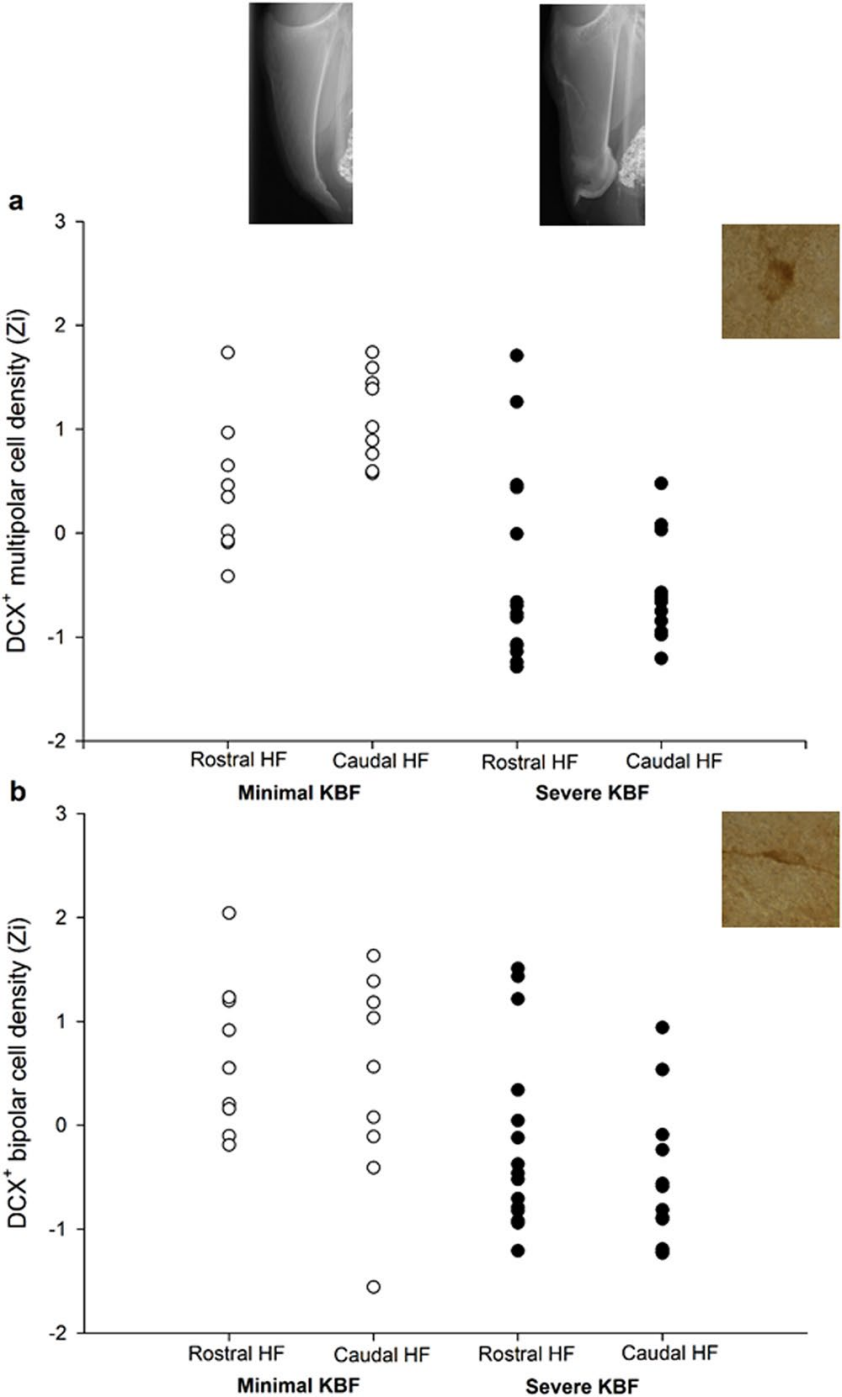
Densities of (**a**) multipolar and (**b**) bipolar DCX-stained cells in the rostral and caudal HF subregions of hens with minimal versus severe keel bone fractures (KBFs) at time point 11, normalised (Zi) for staining batch. Example radiographs reflect the mean severity scores of the minimal and severe KBF groups: 3.5 and 8.5 respectively. Representative DCX^+^ stained cells of both morphologies are displayed at 100X magnification.

Hens with severe KBFs 3–4 weeks before tissue sampling had fewer DCX^+^ multipolar cells than counterparts with minimal fractures in the HF as a whole (*F*_1,14.5_ = 22.56, *p* < 0.001), and there was no effect of HF subregion (*F*_1,20.2_ = 2.93, *p* = 0.102). There was an interaction between subregion and fracture status (*F*_1,20.2_ = 10.52, *p* = 0.004, Fig. 2a), whereby hens with minimal fractures had more DCX^+^ multipolar neurons than hens with severe fractures in both the rostral (*p* = 0.011) and caudal (*p* < 0.001) HF, but the effect was stronger in the caudal HF. Hens with minimal fractures alone had a higher density of stained neurons in the caudal HF than the rostral region (*p* = 0.001), with no subregional difference in hens with severe fractures (*p* = 0.294).

Hens suffering severe KBFs also had fewer DCX^+^ bipolar cells throughout the HF (*F*_1,17.5_ = 12.48, *p* = 0.002). Cell counts did not differ between the rostral and caudal regions (*F*_1, 21.9_ = 3.45, *p* = 0.077), nor did subregion interact with fracture status (*F*_1,21.9_ = 0.069, *p* = 0.796, Fig. 2b).

### Adult hippocampal neurogenesis & KBF severity score

**Figure 3:**
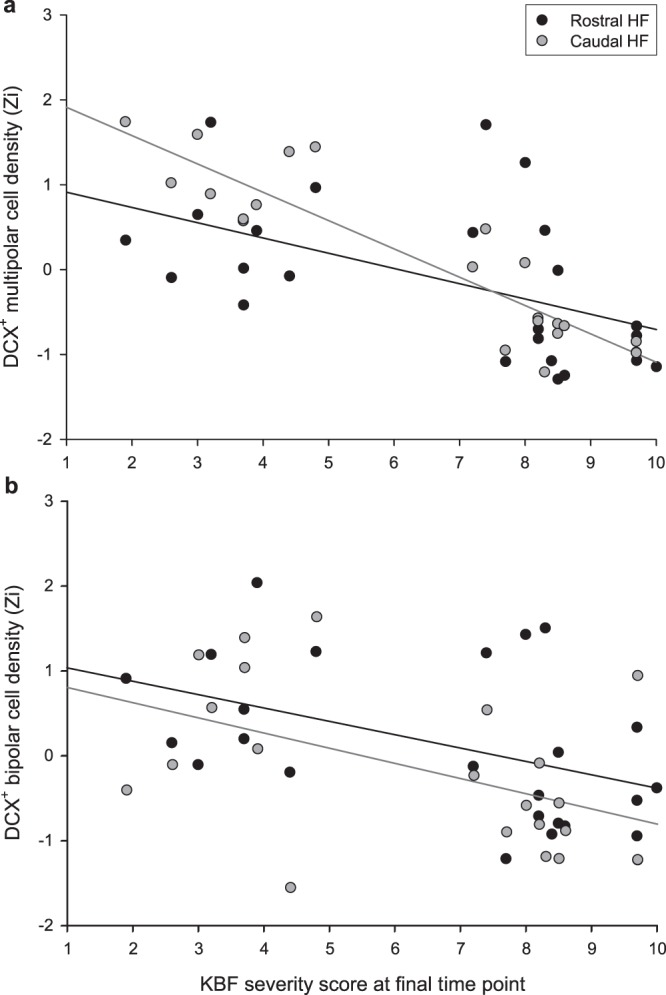
Relationship between keel bone fracture (KBF) score at the final time point (ranging from 0 = no KBF to 10 = extremely severe) and density of DCX-positive (**a**) multipolar and (**b**) bipolar cells in the rostral and caudal HF subregions, normalised (Zi) for staining batch. Simple linear regression lines are plotted for cell densities in the rostral (black) and caudal (grey) HF subregions.

Final KBF severity (3–4 weeks prior to tissue collection) negatively predicted DCX^+^ multipolar cell density across individual birds (*F*_1,16.8_ = 35.98, *p* < 0.001). With severity accounted for, the effect of HF subregion reached significance, with more DCX^+^ multipolar neurons present in the caudal HF (*F*_1,19.8_ = 9.17, *p* = 0.007). KBF severity interacted with subregion, (*F*_1,20.5_ = 7.99, *p* = 0.010), reflecting a steeper relationship between severity score and multipolar cell density in the caudal HF than in the rostral region (Fig. 3a).

Final fracture severity negatively predicted densities of DCX^+^ bipolar neurons (*F*_1,17.4_ = 11.96, *p* = 0.003, Fig. 3b). There was no main effect of HF subregion (*F*_1,21.7_ = 0.460, *p* = 0.505), nor was there an interaction between severity score and subregion (*F*_1,22.3_ = 0.003, *p* = 0.960).

### AHN & activity

Mean number of daily aviary transitions at the final time point (3–4 weeks before tissue sampling) did not predict multipolar DCX^+^ cell densities (*F*_1,14.2_ = 0.271, *p* = 0.611) and did not interact with HF subregion (*F*_1,15.0_ = 0.633, *p* = 0.439). Similarly, mean transitions did not co-vary with density of DCX^+^ bipolar cells (*F*_1,14.3_ = 0.032, *p* = 0.860; transitions*subregion: *F*_1,15.9_ = 0.135, *p* = 0.718).

Furthermore, including transitions as a covariate in LMMs along with KBF severity score did not remove the effect of KBF severity, which continued to negatively predict densities of DCX^+^ multipolar (*F*_1,11.2_ = 22.89, *p* = 0.001) and bipolar (*F*_1,11.5_ = 21.62, *p* = 0.001) cells in the HF.

### Timescale of KBF acquisition

**Figure 4:**
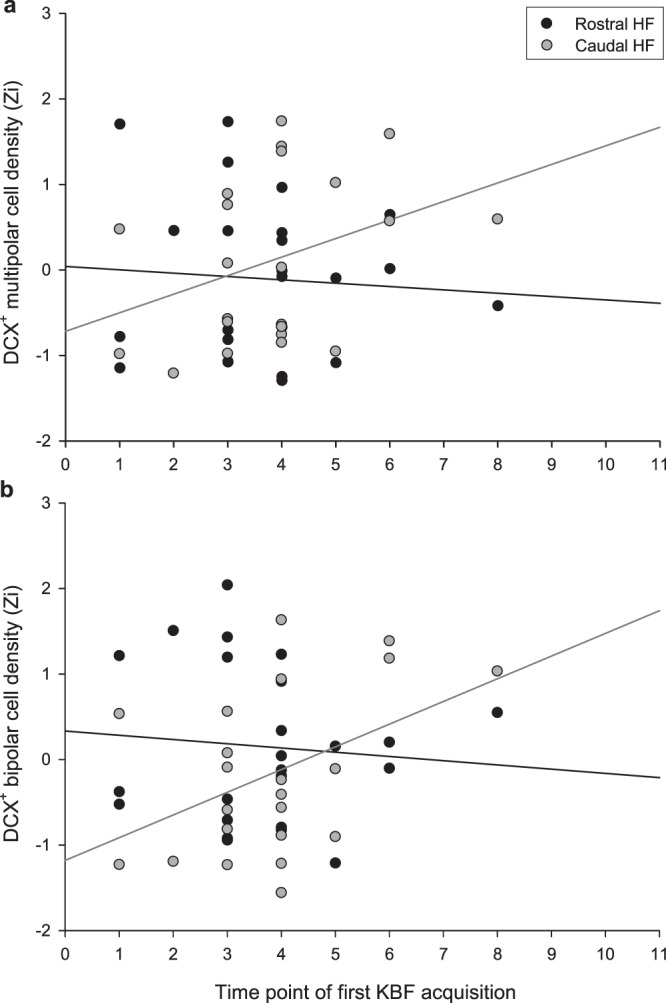
Relationship between the time point at which individual hens first developed a keel bone fracture (KBF) and their density of DCX-positive (**a**) multipolar and (**b**) bipolar cells in the rostral and caudal HF, normalised (Zi) for staining batch. Simple linear regression lines are plotted for cell densities in the rostral (black) and caudal (grey) HF subregions.

For hens that had developed severe KBFs by the final time point (T11), the median time point for acquisition of the first fracture was T3, compared to one time point later (T4) for hens with only minimal damage by the end of the study. Though time point at which a KBF was first acquired did not co-vary with AHN over the whole HF (*F*_1,16.2_ = 1.87, *p* = 0.190), it interacted with subregion to predict DCX^+^ multipolar cell density (*F*_1,20.0_ = 17.63, *p* < 0.001). Specifically, hens that acquired KBFs earlier and thus had suffered them for longer appeared to have fewer new multipolar neurons at the caudal pole (Fig. 4a). A similar effect was observed for bipolar cells (time point *F*_1,20.0_ = 0.267, *p* = 0.611, time point*subregion: *F*_1,21.8_ = 7.33, *p* = 0.013, Fig. 4b).

The time point at which a hen acquired their first KBF was moderately negatively correlated with their fracture severity at the final time point (T11), *r*(22) = −0.561, *p* = 0.004. However, time point of first damage continued to interact with HF subregion to predict caudal DCX^+^ multipolar density when included in the same LMM as KBF severity at T11 (first damage time point*subregion: *F*_1,18.7_ = 7.58, *p* = 0.013; first damage timepoint: *F*_1,13.8_ = 0.649, *p* = 0.434; severity T11: *F*_1,12.0_ = 29.24, *p* < 0.001; severity T11* subregion: *F*_1,18.4_ = 2.189, *p* = 0.156). This was also the case for DCX + bipolar cell density (first damage time point*subregion: *F*_1,20.0_ = 11.10, *p* = 0.003).

LMMs conducted in a stepwise forward manner, with KBF severity at each time point as covariates, confirmed that severity at the final time point (T11) was the best predictor of DCX^+^ multipolar densities over the whole HF at end of life (results reported in section 4.3), with severity at no other time point adding significant explanatory power. Although KBF severity at T11 was the best predictor, when analysing each time point separately, end of life densities of DCX^+^ multipolar cells were predicted by KBF severity scores from time point 4 onwards (T4 severity: *F*_1,17.1_ = 8.11, *p* = 0.011; *p* ≤ 0.001 for severity at all subsequent time points)). DCX^+^ bipolar densities were best predicted by KBF severity at time point 10 (*F*_1,18.1_ = 14.12, *p* = 0.001), and other time points did not add further explanatory power to the model. There was a trend towards bipolar densities being predicted by severity at time point 4, (*F*_1,18.9_ = 3.88, *p* = 0.064), which remained significant for time point 5 onwards (*p* ≤ 0.012).

**Figure 5:**
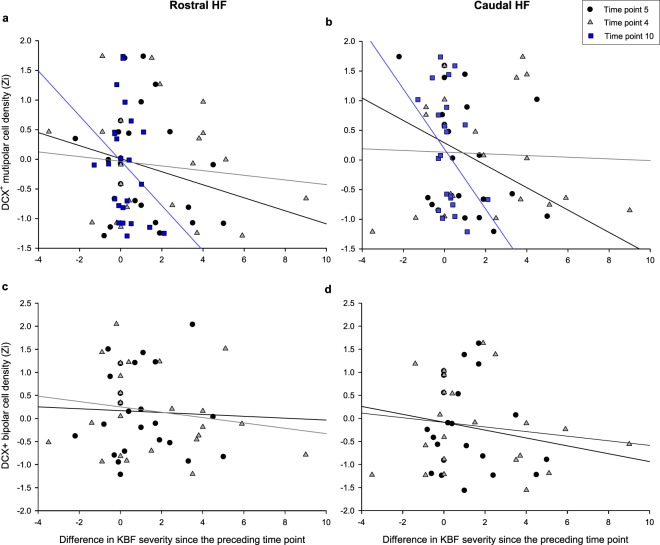
The relationship between the difference in keel bone fracture (KBF) severity score since the preceding time point and densities of DCX^+^ (**a**) rostral multipolar, (**b**) caudal multipolar, (**c**) rostral bipolar and (**d**) caudal bipolar cells, for time points wherein this change in severity predicted cell densities at end of life. Simple linear regression lines are plotted for time points 5 (black), 4 (grey) and 10 (blue).

To explore the integration window over which AHN assimilates changes in the experience of pain/stress, the same stepwise LMM procedure was conducted for the difference in KBF severity at each time point compared to the previous recorded score. Change in KBF severity between time points 4 & 5 (*F*_1,18.9_ = 18.61, *p* < 0.001), 3 & 4 (*F*_1,15.2_ = 6.80, *p* = 0.020) and 9 & 10 (*F*_1,17.1_ = 5.58, *p* = 0.030) negatively predicted DCX^+^ multipolar cell densities at end of life (Fig. 5a,b). Changes in severity between time points 4 & 5 (*F*_1,19.1_ = 13.57, *p* = 0.002) and 3 & 4 (*F*_1,14.7_ = 7.39, *p* = 0.016) similarly predicted DCX^+^ bipolar cell densities, though severity difference between time points 9 & 10 did not (*F*_1,19.4_ = 0.322, *p* = 0.577, Fig. 5c,d).

The predictive power of change in severity between these particular time points did not arise from their contribution to KBF severity at the final time point, as these variables were not correlated (T3 to 4: *r*(22) = 0.187, *p* = 0.381; T4 to 5: *r*(22) = 0.170, *p* = 0.426: T9 to 10: *r*(22) = 0.339, *p* = 0.105).

## Discussion

### Implications for animal welfare

Hens with severe KBFs 3–4 weeks before tissue sampling had lower densities of DCX^+^ multipolar and bipolar neurons in the HF when compared to birds with minimal KBFs, while the magnitude of keel bone injury an individual had sustained by this time also linearly predicted their number of surviving new-born cells. Such downregulated AHN occurs as part of a depression-like physiological state, arising from chronic exposure to stress across numerous paradigms in both mammalian^29,31,76^ and avian^43,45,48,50^ species. As previous work provided behavioural evidence of pain through a place preference for the location of analgesic administration in hens with KBFs^21^, altered HF morphology in terms of reduced AHN in the present study further suggests that the accumulation of fractures is sufficient to lead to long-term stress in affected birds. In mice, a model of chronic neuropathic pain severe enough to suppress AHN was also associated with anxiety- and depressive-like behaviour^32^. The observed neural and affective changes persisted beyond reversal of the injury, indicating that they were not an immediate result of nociceptive stimulation. Given that the hens in the present study showed a similar reduction in AHN following chronic pain, an accompanying depressive-like state may also have been induced. Moreover, the magnitude of keel bone damage present 3–4 weeks before end of life appears to predict the degree of suppression in neural differentiation exhibited, which might also reflect the level of stress experienced. Adult-born neurons in the ventral dentate gyrus of the mouse HF inhibit those mature neurons in the same region that respond preferentially to anxiogenic conditions^77^. Though currently unexplored, if adult-born cells in the chicken HF are similarly involved in inhibiting stress-responsive mature neurons in the caudal subregion, this linear relationship may relate to a cumulative loss of the stress resilience afforded by new neurons with increasing magnitude and/or duration of pain.

Previous research has indicated that it is common for hens to sustain multiple fractures to the keel at different points throughout the laying cycle, with a mean of 3.1 (±1.8 SD) KBFs per hen observed at the end of the laying period in a similar study^78^. Multiple fractures were also observed in the present study, but due to variability in dimensions, location, healing status, etc., quantified KBF severity scores collated all fractures present^71^ (both existing & new), and thus tended to increase incrementally after damage was first sustained. Though it is not known for how long a KBF continues to be a source of pain after acquisition, hens that developed a conditioned place preference for the location of analgesic administration in a previous study had had KBFs for at least three weeks prior to the point of testing^21^, whilst hard callus formation takes four to six weeks to occur^14^. Radiographs in the present study were taken 4 to 5 weeks apart, leading to two possible interpretations of analyses relating the timescale of KBF severity to AHN. The first is based upon the assumption that, after this period of time, previous fractures have healed to the extent that they are no longer painful or stressful, and thus would only have exerted a suppressive effect on AHN during their recorded time point. Therefore, hens with more recent (still healing) fractures should be more affected, which is supported by the finding that severity of most recent KBFs (radiographed 3–4 weeks prior) were the best predictor of AHN. In this case, where hens with older fractures also show corresponding reductions in AHN, the window over which AHN integrates experience must span this length of time. Our results indicate that AHN at end of life is predicted by KBF severity from around time point 4 onwards, meaning AHN would thus integrate experience from around 34 weeks previously.

The alternative interpretation is based on the assumption that healed breaks remain somewhat painful, and therefore stressful, over a hen’s lifetime. Indeed, existing KBFs may often be repeatedly aggravated during normal hen behaviour, such as movement to food or water, flight and perching^15^. If this is the case, through integrating older and newer fractures, severity score at the final time point in itself reflects the cumulative pain or discomfort experienced. This means it is not possible to determine the time scale over which AHN integrates painful experience, as that occurring in the past persists until the end of life. As such, the contribution of recent versus longer-term pain or stress to the relationship between AHN and KBF severity measured at the latest point (3–4 weeks previously) cannot be distinguished. However, in the caudal HF subregion, a relationship existed between AHN and the duration of time since fractures were first incurred. Moreover, this variable predicted caudal AHN independently of severity at the final time point. Furthermore, changes in KBF severity score since the preceding time point occurring as early as ~37 weeks previously also predicted AHN over the whole HF, and were not correlated with final fracture severity. These relationships were not as strong as those with end of life KBF severity, suggesting that AHN may integrate temporally distant experience on a more subtle level, alongside that which is most recent or cumulative. The finding that the caudal HF is more sensitive to certain aspects of this integration are consistent with its hypothesised greater responsivity to chronic stress^60^. Before future studies definitively elucidate the time scale over which KBFs cause pain, it is not possible to know which assumption is correct, meaning the results from the present study cannot determine the window of time over which AHN integrates negative experience.

Though individual differences in activity levels masked differences between minimal and severe KBF hens in the absolute number of vertical aviary transitions made at the final time point, hens that had developed severe KBFs by this point made fewer transitions than they had at the first time point, before most individuals had suffered any damage. This indicates that birds with severe KBFs, as selected for the present sample, show a corresponding reduction in activity. The assumption that it is painful for them to move is consistent with reported analgesic-driven reductions in latency for hens with KBFs to descend from raised perches^18^. On the other hand, lower AHN in hens with severe fractures could not be explained by their recorded activity 3–4 weeks before tissue sampling, and thus cannot be attributed to a consequence of varying levels of activity or concurrent spatial cognitive processing at this time. Our findings thus support the inference that hens with KBFs experience a corresponding negative affective state beyond an acute sensory pain response. Since this is likely to influence overall quality of life for laying hens in commercial systems, management steps to reduce or delay acquisition of KBFs are likely to have a notable impact on welfare.

### Hippocampal homologies

In the HF, hens with severe KBFs 3–4 weeks prior to tissue collection had lower densities of DCX^+^ multipolar and bipolar neurons when compared to birds with minimal KBFs. These findings are consistent with the suppressive effect of induced chronic neuropathic pain on AHN in mice^32^ and suggest a homologous response to prolonged pain in the avian HF. In line with the recent demonstration of reduced AHN in hens exposed to unpredictable chronic mild stress^50^, the current findings support the conclusion that the regulation of AHN in modern mammals and birds derives from a process established in their common ancestor over 300 million years ago^79^.

In rodents, some models of chronic stress have a ventral-specific influence on AHN, whereas others result in suppression across the whole dentate gyrus (see O’Leary and Cryan^29^ for a review). Whether the effect is regionally-localised may depend on the nature of the stressor or paradigm employed. In the present study, KBFs were associated with suppressed AHN over the whole laying hen HF, rather than being restricted to the putatively more stress-sensitive caudal region^60^. In a mouse model, Dimitrov *et al*.^32^ also observed a qualitatively similar reduction in AHN in the dorsal and ventral subregions of the HF following induced chronic pain. Therefore, whereas a sequence of mild, albeit unpredictable stressors (as in Gualtieri *et al*.^50^) appears to produce a caudal-specific suppression of laying hen AHN, sources of chronic pain such as KBFs may conceivably present stressors of greater magnitude, which affect neurogenesis across more of the HF.

That being said, for multipolar DCX^+^ neurons, the interaction between individual KBF scores and HF subregion demonstrates that the suppressive effect of these fractures on AHN is stronger at the caudal pole. Moreover, the caudal HF appears more sensitive to temporal aspects of KBF acquisition, with the relationship between AHN and the duration of time since fractures were first incurred only existing in the caudal region.

Accounting for fracture severity scores also revealed an overall greater density of new multipolar neurons in the caudal HF than the rostral subregion, as found in our lab previously^50^. This regional gradient has been observed in certain other avian species, with the same pattern present in zebra finches^80^ and two species of North American blackbird^81^. However, adult wild-caught chickadees instead have a higher level of AHN in the rostral HF subregion. Interestingly though, this gradient is eliminated after 6 weeks in captivity^45^. When comparing KBF groups in the present study, hens with minimal fractures alone had more new multipolar neurons in the caudal HF than the rostral region, whereas hens with severe KBFs exhibited no subregional difference. Though further exploration is required, it could be that sources of chronic stress, such as KBFs and captivity, have some form of homogenising effect on subregional AHN rates.

In general, the results obtained were qualitatively similarly for multipolar and bipolar/fusiform cells, assumed to be respectively more mature versus younger and still migrating^74^, though relationships with KBF severity were stronger for the multipolar cells. This may suggest that the chronic stress arising from severe KBFs particularly affects later neuronal cell survival. The relative impact of stress on various stages of AHN appears to differ between paradigms^82^, but psychosocial stress has similarly been reported to downregulate the longer-term survival, but not the proliferation, differentiation or immediate survival, of newly-generated cells in the rat HF^83,84^.

In contrast to fracture severity, the number of transitions hens made between aviary zones 3–4 weeks before tissue sampling was not associated with AHN when employed as a proxy for activity. Such a relationship might have been predicted based on rodent studies, wherein time spent voluntarily running in a wheel correlates positively with AHN^64,85^. However, flight exercise was previously found not to be associated with DCX-expression in starlings^86^. Infrared tracking in the present study also recorded only vertical activity within the aviary, meaning individual differences in activity within a single horizontal zone or tier were not accounted for. Additionally, while voluntary exercise robustly upregulates AHN in rodents, forced exercise does not have this stimulatory effect^35^. Some transitions recorded in the present study may have equated to forced movement, as hens evaded people entering the aviary or were displaced by flock-mates during competition for space or food, with injured hens perhaps particularly affected. Involuntary activity may as such have contributed to the lack of observed relationship. For these reasons, further exploration into the association between exercise and AHN in avian species may be required in future. It would also be worthwhile to consider more complex assessments of hen movement, that account for consistency and changes in patterns over multiple days and longer periods of a hens’ life (as in Rufener, *et al*.^65^).

## Conclusions

In commercial laying hens, severe KBFs present 3–4 weeks before sampling are associated with a reduced density of new-born neurons across the HF when compared to flock-mates with minimal KBFs. Additionally, KBF severity scores at this time negatively predict DCX^+^ cell counts across individual hens, while in the caudal HF, hens that developed KBFs earlier tend to have fewer DCX^+^ multipolar neurons. Further information regarding the duration that KBFs continue to be a source of pain or stress will be necessary in order to determine the time scale in which AHN over the whole HF integrates this painful experience. On the other hand, vertical movement within the aviary 3–4 weeks prior to sampling is not associated with AHN levels. Results suggest that, like the rodent hippocampus, the avian HF is sensitive to the experience of pain as a result of KBFs, on a time scale of at least 3–4 weeks. Downregulated AHN lends support to the notion that KBFs present a source of chronic stress, which is associated with induction of a depression-like state in mammals and thus likely detrimental to the affective experience of commercial laying hens. Management steps to reduce or delay acquisition of KBFs in commercial laying hen systems are thus likely to have a notable impact on animal welfare.

## Data Availability

The datasets generated during and analysed during the current study are available from the corresponding author on reasonable request.

## References

[1] Heerkens, J. L. T. *et al*. In *9th European Poultry Conference*. (eds. R. Tauson, H. J. Blokhuis, L. Berg, & A. Elson) (2013).

[2] Petrik MT, Geurin MT, Widowski TM (2015). On-farm comparison of keel fractureprevalence and other welfare indicators in conventional cage and floor-housed laying hens in Ontario, Canada. Poultry Science.

[3] Riber AB, Hinrichsen LK (2016). Keel-bone damage and foot injuries in commercial laying hens in Denmark. Animal Welfare.

[4] Rodenburg TB (2008). Welfare assessment of laying hens in furnished cages and non-cage systems: an on-farm comparison. Animal Welfare.

[5] Kappeli S, Gebhardt-Henrich SG, Frohlich E, Pfulg A, Stoffel MH (2011). Prevalence of keel bone deformities in Swiss laying hens. British Poultry Science.

[6] Stratmann Ariane, Fröhlich Ernst Konrad Friederich, Gebhardt-Henrich Sabine Gabriele, Harlander-Matauschek Alexandra, Würbel Hanno, Toscano Michael Jeffrey (2015). Modification of aviary design reduces incidence of falls, collisions and keel bone damage in laying hens. Applied Animal Behaviour Science.

[7] Wilkins LJ (2011). Influence of housing system and design on bone strength and keel bone fractures in laying hens. Veterinary Record.

[8] Tarlton JF, Wilkins LJ, Toscano MJ, Avery NC, Knott L (2013). Reduced bonebreakage and increased bone strength in free range laying hens fed omega-3 polyunsaturated fatty acidsupplemented diets. Bone.

[9] Toscano MJ (2015). The effects of long (C20/22) and short (C18) chain omega-3 fatty acids on keelbone fractures, bone biomechanics, behaviour and egg production in free range laying hens. Poultry Science.

[10] Richards GJ (2012). Pop hole use by hens with different keel fracture status monitored throughout the laying period. Veterinary Record.

[11] Sandilands V, Moinard C, Sparks NH (2009). Providing laying hens with perches: fulfilling behavioural needs but causing injury?. Bristish Poultry Science.

[12] Weeks CA, Nicol CJ (2006). Behavioural needs, priorities and preferences of laying hens. World’s Poultry Science Journal.

[13] Gebhardt-Henrich SG, Fröhlich EKF (2015). Early onset of laying and bumblefoot favor keel bone fractures. Animals.

[14] Richards GJ (2011). Continuous monitoring of pop hole usage by commercially housed free-range hens throughout the production cycle. Veterinary Record.

[15] Riber AB, Casey-Trott TM, Herskin MS (2018). The Influence of Keel Bone Damage on Welfare of Laying Hens. Frontiers in Veterinary Science.

[16] Rufener C (2019). Keel bone fractures are associated with individual mobility of laying hens in an aviary system. Applied Animal Behaviour Science.

[17] Rentsch AK, Rufener CB, Spadavecchia C, Stratmann A, Toscano MJ (2019). Laying hen’s mobility is impaired by keel bone fractures and does not improve with paracetamol treatment. Applied Animal Behaviour Science.

[18] Nasr MAF, Nicol CJ, Murrell JC (2012). Do Laying Hens with Keel Bone Fractures Experience Pain?. PLoS ONE.

[19] Nasr MA, Nicol CJ, Wilkins L, Murrell JC (2015). The effects of two non-steroidal anti-inflammatory drugs on the mobility of laying hens with keel bone fractures. Veterinary Anaesthesia and Analgesia.

[20] Nasr MAF, Murrell J, Wilkins LJ, Nicol CJ (2012). The effect of keel fractures on egg-production parameters, mobility and behaviour in individual laying hens. Animal Welfare.

[21] Nasr MAF (2013). Positive affective state induced by opioid analgesia in laying hens with bone fractures. Applied Animal Behaviour Science.

[22] Herman JP (2016). Regulation of the hypothalamic-pituitary-adrenocortical stress response. Comprehensive Physiology.

[23] McEwen BS (1998). Stress, adaptation, and disease. Allostasis and allostatic load. Annals of the New York Academy of Sciences.

[24] El-Lethey H, Huber-Eicher B, Jungi TW (2003). Exploration of stress-induced immunosuppression in chickens reveals both stress-resistant and stress-susceptible antigen responses. Veterinary Immunology and Immunopathology.

[25] Shini S, Shini A, Huff GR (2009). Effects of chronic and repeated corticosterone administration in rearing chickens on physiology, the onset of lay and egg production of hens. Physiology & Behavior.

[26] Poirier CBM (2019). Validation of hippocampal biomarkers of cumulative affective experience. Neuroscience & Biobehavioral Reviews.

[27] Altman J, Das GD (1965). Autoradiographic and histological evidence of postnatal hippocampal neurogenesis in rats. Journal of Computational Neurology.

[28] van Praag H (2002). Functional neurogenesis in the adult hippocampus. Nature.

[29] O’Leary OF, Cryan JF (2014). A ventral view on antidepressant action: roles for adult hippocampal neurogenesis along the dorsoventral axis. Trends in Pharmacological Science.

[30] Warner-Schmidt JL, Duman RS (2006). Hippocampal neurogenesis: opposing effects of stress and antidepressant treatment. Hippocampus.

[31] Gould E, Tanapat P, McEwen BS, Flügge G, Fuchs E (1998). Proliferation of granule cell precursors in the dentate gyrus of adult monkeys is diminished by stress. Proceedings of the National Academy of Sciences of the United States of America.

[32] Dimitrov EL, Tsuda MC, Cameron HA, Usdin TB (2014). Anxiety- and Depression-Like Behavior and Impaired Neurogenesis Evoked by Peripheral Neuropathy Persist following Resolution of Prolonged Tactile Hypersensitivity. The Journal of Neuroscience.

[33] Kempermann G, Kuhn HG, Gage FH (1997). More hippocampal neurons in adult mice living in an enriched environment. Nature.

[34] Jin J (2008). Voluntary exercise increases the new cell formation in the hippocampus of ovariectomized mice. Neuroscience Letters.

[35] Van Praag H, Kempermann G, Gage FH (1999). Running increases cell proliferation and neurogenesis in the adult mouse dentate gyrus. Nature Neuroscience.

[36] Manev H, Uz T, Smalheiser NR, Manev R (2001). Antidepressants alter cell proliferation in the adult brain *in vivo* and in neural cultures *in vitro*. European Journal of Pharmacology.

[37] Malberg JE (2000). Chronic Antidepressant Treatment Increases Neurogenesis in Adult Rat Hippocampus. Journal of Neuroscience.

[38] Jayatissa MN, Bisgaard C, Tingstrom A, Papp M, Wiborg O (2006). Hippocampal cytogenesis correlates to escitalopram-mediated recovery in a chronic mild stress rat model of depression. Neuropsychopharmacology.

[39] Santarelli L (2003). Requirement of Hippocampal Neurogenesis for the Behavioural Effects of Antidepressants. Science.

[40] Surget A (2011). Antidepressants recruit new neurons to improve stress response regulation. Molecular Psychiatry.

[41] Schloesser RJ, Lehmann M, Martinowich K, Manji HK, Herkenham M (2010). Environmental enrichment requires adult neurogenesis to facilitate the recovery from psychosocial stress. Molecular Psychiatry.

[42] Melleu FF, Pinheiro MV, Lino-de-Oliveira C, Marino-Neto J (2016). Defensive behaviours and prosencephalic neurogenesis in pigeons (*Columba livia*) are affected by environmental enrichment in adulthood. Brain Structure & Function.

[43] LaDage LD, Roth TC, Fox RA, Pravosudov VV (2010). Ecologically-relevant spatial memory use modulates hippocampal neurogenesis. Proceedings of the Royal Society B.

[44] Patel SN, Clayton NS, Krebs JR (1997). Spatial learning induces neurogenesis in the avian brain. Behavioural Brain Research.

[45] Barnea A, Nottebohm F (1994). Seasonal recruitment of hippocampal neurons in adult free-ranging black-capped chickadees. Proceedings of the National Academy of Sciences USA.

[46] Roth TC, LaDage LD, Freas CA, Pravosudov VV (2012). Variation in memory and the hippocampus across populations from different climates: a common garden approach. Proceedings of the Royal Society B.

[47] Barnea A, Pravosudov VV (2011). Birds as a model to study adult neurogenesis: bridging evolutionary, comparative and neuroethological approaches. European Journal of Neuroscience.

[48] Robertson B (2017). Food restriction reduces neurogenesis in the avian hippocampal formation. PLoS ONE.

[49] Taufique SKT, Prabhat A, Kumar V (2018). Constant light environment suppresses maturation and reduces complexity of new born neuron processes in the hippocampus and caudal nidopallium of a diurnal corvid: Implication for impairment of the learning and cognitive performance. Neurobiology of Learning and Memory.

[50] Gualtieri F (2019). Unpredictable Chronic Mild Stress Suppresses the Incorporation of New Neurons at the Caudal Pole of the Chicken Hippocampal Formation. Scientific Reports.

[51] Fanselow MS, Dong H (2010). Are The Dorsal and Ventral Hippocampus functionally distinct structures?. Neuron.

[52] Zhang WN, Pothuizen HH, Feldon J, Rawlins JN (2004). Dissociation of function within the hippocampus: effects of dorsal, ventral and complete excitotoxic hippocampal lesions on spatial navigation. Neuroscience.

[53] Bannerman DM (2004). Regional dissociations within the hippocampus–memory and anxiety. Neuroscience Biobehavioural Reviews.

[54] Moser MB, Moser EI (1998). Functional differentiation in the hippocampus. Hippocampus.

[55] Sahay A, Hen R (2007). Adult hippocampal neurogenesis in depression. Nature Neuroscience.

[56] Tanti A, Rainer Q, Minier F, Surget A, Belzung C (2012). Differential environmental regulation of neurogenesis along the septo-temporal axis of the hippocampus. Neuropharmacology.

[57] Hawley DF, Leasure JL (2012). Region-specific response of the hippocampus to chronic unpredictable stress. Hippocampus.

[58] Hawley DF, Morch K, Christie BR, Leasure JL (2012). Differential response of hippocampal subregions to stress and learning. PLoS ONE.

[59] Colombo M, Broadbent N (2000). Is the avian hippocampus a functional homologue of the mammalian hippocampus?. Neuroscience Biobehavioural Reviews.

[60] Smulders TV (2017). The avian hippocampal formation and the stress response. Brain, Behaviour & Evolution.

[61] Bouillé C, Baylé JD (1973). Effects of limbic stimulations or lesions on basal and stress induced hypothalamic pituitary adrenocortical activity in the pigeon. Neuroendocrinology.

[62] Couillard-Despres S (2005). Doublecortin expression levels in adult brain reflect neurogenesis. European Journal of Neuroscience.

[63] Balthazart J, Boseret G, Konkle AT, Hurley LL, Ball GF (2008). Doublecortin as a marker of adult neuroplasticity in the canary song control nucleus HVC. European Journal of Neuroscience.

[64] Allen DM (2001). Ataxia telangiectasia mutated is essential during adult neurogenesis. Genes & Development.

[65] Rufener C (2018). Finding hens in a haystack: Consistency of movement patterns within and across individual laying hens maintained in large groups. Scientific Reports.

[66] Siegford JM (2016). Assessing Activity and Location of Individual Laying Hens in Large Groups Using Modern Technology. Animals.

[67] Jones RB (1992). The nature of handling immediately prior to test affects tonic immobility fear reactions in laying hens and broilers. Applied Animal Behaviour Science.

[68] Buijs S (2018). Behavioural and physiological responses of laying hens to automated monitoring equipment. Applied Animal Behaviour Science.

[69] Širovnik, J. & Toscano, M. J. Restraining laying hens for radiographic diagnostics of keel bones. In *Proceedings of the 10th European Symposium on Poultry Welfare,* 162 (2017).

[70] Rufener C, Baur S, Stratmann A, Toscano MJ (2018). Keel bone fractures affect egg laying performance but not egg quality in laying hens housed in a commercial aviary system. Poultry Science.

[71] Rufener C, Baur S, Stratmann A, Toscano MJ (2018). A reliable method to assess keel bone fractures in laying hens from radiographs using a tagged visual analogue scale. Frontiers in Veterinary Science.

[72] Fabel K (2009). Additive effects of physical exercise and environmental enrichment on adult hippocampal neurogenesis in mice. Frontiers in Neuroscience.

[73] Puelles, L. *The chick brain in stereotaxic coordinates: an atlas featuring neuromeric subdivisions and mammalian homologies*., (Boston: Academic Press, 2007).

[74] Boseret G, Ball GF, Balthazart J (2007). The microtubule-associated protein doublecortin is broadly expressed in the telencephalon of adult canaries. Journal of Chemical Neuroanatomy.

[75] Tömböl, T., Davies, D. C., Németh, A., Sebestény, T. & Alpár, A. A comparative Golgi study of chicken (*Gallus domesticus*) and homing pigeon (*Columba livia*) hippocampus. *Anatomical Embryology* **201**, 85-101 (2000). 10.1007/pl00008235 10672361

[76] Gould E, Tanapat P (1999). Stress & Hippocampal Neurogenesis. Biological Psychiatry.

[77] Anacker C (2018). Hippocampal neurogenesis confers stress resilience by inhibiting the ventral dentate gyrus. Nature.

[78] Baur, S., Rufener, C., Toscano, M. J. & Geissbühler, U. Radiographic evaluation of keel bone damage in laying hens – morphologic and temporal observations in a longitudinal study. *Frontiers in Veterinary Science* (in press). 10.3389/fvets.2020.00129 PMC7081720 32226794

[79] Striedter GF (2016). Evolution of the Hippocampus in Reptiles and Birds. The Journal of Comparative Neurology.

[80] Barnea A, Mishal A, Nottebohm F (2006). Social and spatial changes induce multiple survival regimes for new neurons in two regions of the adult brain: An anatomical representation of time?. Behavioural Brain Research.

[81] Guigueno MF, MacDougall-Shackleton SA, Sherry DF (2016). Sex and seasonal differences in hippocampal volume and neurogenesis in brood-parasitic brown-headed cowbirds (*Molothrus ater*). Developmental Neurobiology.

[82] Jorgensen C, Taylor J, Barton T (2019). The Impact of Ethologically Relevant Stressors on Adult Mammalian Neurogenesis. Brain Sciences.

[83] Thomas RM, Hotsenpiller G, Peterson DA (2007). Acute Psychosocial Stress Reduces Cell Survival in Adult Hippocampal Neurogenesis without Altering Proliferation. Journal of Neuroscience.

[84] Buwalda B, Van Der Borght K, Koolhaas JM, McEwen BS (2010). Testosterone decrease does not play a major role in the suppression of hippocampal cell proliferation following social defeat stress in rats. Physiology & Behavior.

[85] Rhodes JS (2003). Exercise increases hippocampal neurogenesis to high levels but does not improve spatial learning in mice bred for increased voluntary wheel running. Behavioral Neuroscience.

[86] Hall ZJ (2014). Site-specific regulation of adult neurogenesis by dietary fatty acid content, vitamin E and flight exercise in European starlings. European Journal of Neuroscience.

